# A novel prognostic risk score model based on immune-related genes in patients with stage IV colorectal cancer

**DOI:** 10.1042/BSR20201725

**Published:** 2020-10-23

**Authors:** Ke Xu, Jie He, Jie Zhang, Tao Liu, Fang Yang, Tao Ren

**Affiliations:** 1 Department of Oncology, Clinical Medical College and The First Affiliated Hospital of Chengdu Medical College, Chengdu, Sichuan, People’s Republic of China; 2 Department of Pulmonary and Critical Care Medicine, Clinical Medical College and The First Affiliated Hospital of Chengdu Medical College, Chengdu, Sichuan, People’s Republic of China

**Keywords:** immune-related genes, metastatic colorectal cancer, overall survival, prognostic model, risk score

## Abstract

Purpose: The aims of the present study were to explore immune-related genes (IRGs) in stage IV colorectal cancer (CRC) and construct a prognostic risk score model to predict patient overall survival (OS), providing a reference for individualized clinical treatment.

Methods: High-throughput RNA-sequencing, phenotype, and survival data from patients with stage IV CRC were downloaded from TCGA. Candidate genes were identified by screening for differentially expressed IRGs (DE-IRGs). Univariate Cox regression, LASSO, and multivariate Cox regression analyses were used to determine the final variables for construction of the prognostic risk score model. GSE17536 from the GEO database was used as an external validation dataset to evaluate the predictive power of the model.

Results: A total of 770 candidate DE-IRGs were obtained, and a prognostic risk score model was constructed by variable screening using the following 12 genes: *FGFR4, LGR6, TRBV12-3, NUDT6, MET, PDIA2, ORM1, IGKV3D-20, THRB, WNT5A, FGF18*, and *CCR8*. In the external validation set, the survival prediction C-index was 0.685, and the AUC values were 0.583, 0.731, and 0.837 for 1-, 2- and 3-year OS, respectively. Univariate and multivariate Cox regression analyses demonstrated that the risk score model was an independent prognostic factor for patients with stage IV CRC. High- and low-risk patient groups had significant differences in the expression of checkpoint coding genes (ICGs).

Conclusion: The prognostic risk score model for stage IV CRC developed in the present study based on immune-related genes has acceptable predictive power, and is closely related to the expression of ICGs.

## Introduction

Colorectal cancer (CRC) ranks third for both morbidity and mortality among cancers worldwide [1]. The main cause of death in patients with CRC is distant metastasis. Currently, most CRC patients (approximately 50–60%) have stage IV disease at initial diagnosis [2–4]. Remarkably, stage IV CRC has a better prognosis, relative to other metastatic gastrointestinal malignancies [1]. At present, both the European Society for Medical Oncology (ESMO) and the National Comprehensive Cancer Network (NCCN) have established specific clinical practice guidelines for patients with stage IV CRC and recommended the stratified management of such patients with different disease characteristics, typifying current connotation of ‘precision medicine’. CRC is a heterogeneous disease, and prognosis varies significantly among patients. Thus, development of prognostic risk models can further improve treatment strategies for stratified management; however, most existing prognostic risk models were constructed based on data from patients with CRC overall or those with postoperative stage II CRC [5–8]. Hence, it is necessary to establish a prognostic prediction model for individual with stage IV CRC.

Malignant tumors can induce immune tolerance through a variety of mechanisms, as well as evading immune killing, leading to disease progression and directly affecting patient prognosis. In the process of immune tolerance induction, immune-related genes (IRGs) expressed by tumor cells directly influence the functions of various immune cells [9,10]. In CRC, some IRGs are confirmed to affect the development of tumor cells via immune regulation, such as FAP, high expression of FAP can lead to enrichment of macrophages, monocytes, and Tregs, as well as depletion of Th1 cells and natural killer T cells, generating to an immunosuppressive environment in CRC cells [11]. In another example, high expression of SMAD7 are associated with an inflamed gut, with immune cells infiltration that can elicit antitumor responses, thereby inhibiting CRC cell growth [12]. Hence, construction of a tumor prognostic model based on IRGs is a robust, widely recognized approach; however, no such study has been conducted for stage IV CRC. Therefore, in the present study we mined for IRGs involved in stage IV CRC and constructed a prognostic risk score model based on the identified genes.

## Materials and methods

### Data acquisition

RNA sequencing gene expression data (from 136 tissues, including 85 tumor and 51 normal tissues; workflow type, HTSeqFPKM) and corresponding clinical information (from 85 stage IV CRC cases) were downloaded from The Cancer Genome Atlas (TCGA) official website (https://portal.gdc.cancer.gov/) on April 1, 2020. Gene array and survival data from 38 patients with stage IV CRC (dataset GSE17536) were downloaded from Gene Expression Omnibus (GEO) (https://www.ncbi.nlm.nih.gov/geo/) on April 2, 2020. A gene set, including 1811 IRGs, was downloaded from the ImmPort database (https://immport.niaid.nih.gov/home). An immune checkpoint coding genes (ICGs) set, containing 47 genes, was constructed based on a literature review (Supplementary Table S1).

### Screening of candidate genes, Gene Ontology (GO) term enrichment analysis, Kyoto Encyclopedia of Genes and Genomes (KEGG) pathway analysis, protein–protein interaction (PPI) network analysis, and identification of core genes

The limma package was used to analyze differentially expressed genes (DEGs) between tumor and paracancer tissue samples from TCGA [13]. Genes with |log2 fold changes (FC)| ≥ 1 and adjusted *P*<0.05 were considered as significantly DEGs. Candidate differentially expressed IRGs (DE-IRGs) were obtained by taking the intersection of DEGs and IRGs. The Ggplot2, enrichplot, and clusterprofiler packages were used to perform GO and KEGG analyses, with candidate DE-IRGs as input [14]. GO analysis included three parts: cellular component (CC), molecular function (MF), and biological process (BP). PPI Network analysis was performed by inputting candidate DE-IRGs into the STRING database (https://string-db.org/) [15]. A value of 0.7 was set as the cutoff for confidence scores. Cytoscape software was used to further analyze the resulting PPI network [16]. Finally, genes with higher node degrees were identified as hub genes.

### Establishment and verification of prognostic risk score model

Two datasets, including data from 85 and 38 patients with stage IV CRC from TCGA and GSE17536, were used as the training and verification sets, respectively, for establishment of the prognostic risk score model. In the training set, univariate Cox, LASSO Cox, and multivariate regression analyses were used to select the minimum gene subset [17]. Multivariate regression analysis was performed using the stepwise method. The prognostic score model formula, established after the identification of variables and risk score calculation, was as follows:

Risk score = β1 × Expβ1 + β2 × Expβ2 + β3 × Expβ3 + βi × Expβi

The model was validated using the validation set. The survminer package was used for survival analysis of patients in the high- and low-risk groups, while the survival ROC package was used to construct time dependent receiver operating characteristic (ROC) curves and calculate the area under the ROC curve (AUC) at 1-, 2-, and 3-year overall survival (OS). Univariate and multivariate Cox regression survival analyses were performed by adding the factor of risk score in the training set.

### Immune-related characteristics of high- and low-risk patients based on the prognostic risk score model

Data from 85 patients with stage IV CRC patients from TCGA were divided into high- and low-risk groups by setting the median risk score as the cut-off value. The CIBERSORT algorithm was used to analyze the proportion of 22 immune cells types in tumor tissue samples from the two groups [18]. A Mann–Whitney test was used to evaluate the significance of differences in the composition of the 22 immune cells types and expression levels of immune checkpoint coding genes (ICGs) between the high- and low-risk groups. Pairwise relationships between the expression levels of ICGs were analyzed using Spearman correlation analyses in the ggcorrplot package. *P*<0.05 was considered statistically significant. Finally, the TISIDB database (http://cis.hku.hk/TISIDB/index.php) was employed to analyze the relationship between clinical outcomes and expression levels of the 12 genes included in the prognostic risk score model [19].

### Statistical analysis

R software v3.6.1 (www.r-project.org) was used for statistical analyses in the present study. Numerical variables were described as means ± SD. The Kaplan–Meier method was used for survival analysis, and the log-rank test applied to evaluate differences between groups. Mann–Whitney test was used for nonparametric comparisons. All statistical tests were two-tailed; *P*<0.05 was considered to indicate a significant difference.

## Results

### Clinical characteristics of patients with stage IV CRC

**Figure 1:**
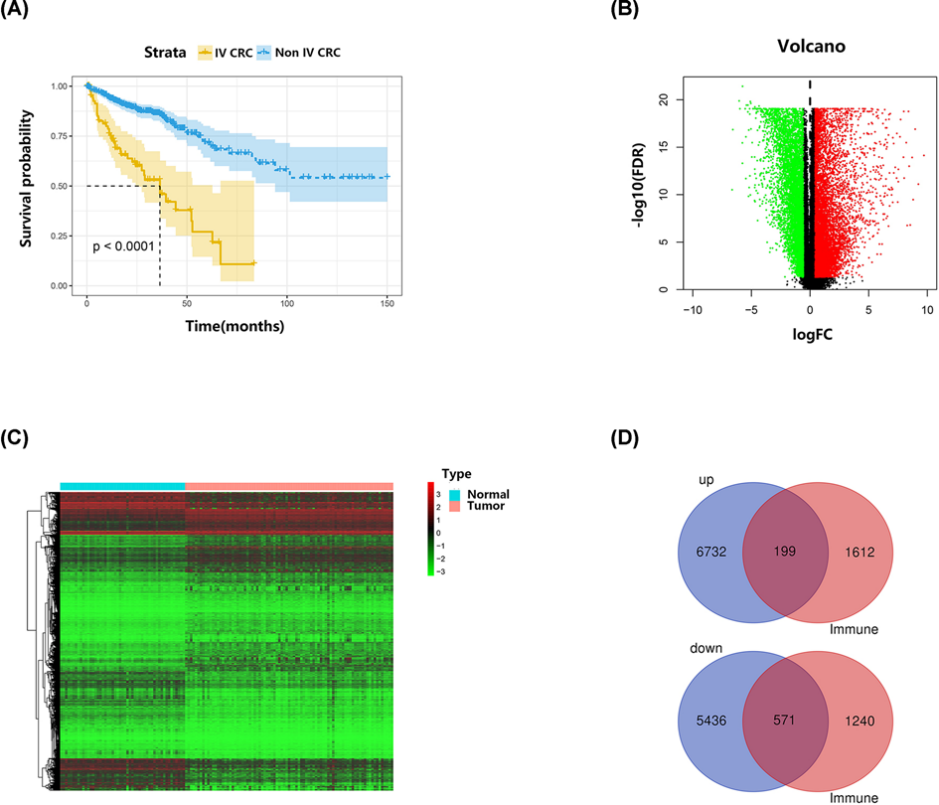
The survival analysis of CRC patients and identification of candidate DE-IRGs (**A**) Kaplan–Meier survival curves of the stage IV CRC patients and the remaining 493 non-stage IV patients from TCGA. (**B**) The volcano figure of DEGs between tumor tissues and normal tissues. (**C**) The heatmap of DEGs between tumor tissues and normal tissues. (**D**) The venn diagram of significantly up-regulated and down-regulated candidate DE-IRGs in intersection of DEGs and IRGs.

Analyzed clinical characteristics of patients with stage IV CRC from TCGA included age, sex, ethnicity, primary site, carcinoembryonic antigen level (CEA), T stage, N stage, overall survival, and survival status. Among the 85 patients with stage IV CRC with complete clinical information in TCGA, mean age was 64.05 ± 12.82 years, the proportion of males was 58.82%, and median OS was 16.8 months. As shown in Figure 1A, there was a significant difference in survival between the 85 patients with stage IV CRC and the remaining 493 patients with non-stage IV disease in TCGA (*P*<0.0001). The 38 patients with stage IV CRC from GSE17536 had an average age of 64.55 ± 11.08 years, 65.79% were male, and median OS was 16.45 months. Details for each clinical parameter are presented in Table 1.

**Table 1:** The clinical characteristics of IV CRC patients

Clinical characteristic	TCGA		GSE17536	
	Total (*n*=85)	Percent (%)	Total (*n*=38)	Percent (%)
**Age**				
<60	32	37.65	14	36.84
≥60	53	62.35	24	63.16
**Gender**				
Male	50	58.82	25	65.79
Female	35	41.18	13	34.21
**Race**				
White	55	64.71	33	86.84
Non-White	30	35.29	5	13.16
**Site**				
Right	23	27.06	NA	NA
Left	33	38.82	NA	NA
Rectum	14	16.47	NA	NA
Unknown	15	17.65	NA	NA
**CEA**				
≤5 ng/ml	12	14.12	NA	NA
>5 ng/ml	48	56.47	NA	NA
Unknown	25	29.41	NA	NA
**T stage**				
T1	0	0	NA	NA
T2	2	2.35	NA	NA
T3	56	65.88	NA	NA
T4	27	31.77	NA	NA
**N stage**				
0	10	11.76	NA	NA
1	34	40.00	NA	NA
2	41	48.24	NA	NA
**Survival status**				
Living	45	52.94	10	26.32
Dead	40	47.06	28	73.68

Abbreviations: CEA, carcinoembryonic antigen; NA, not available.

### Identification of candidate IRGs for stage IV CRC

Comparison of gene expression levels between tumor and paracancer tissue samples identified a total of 12,938 DEGs, including 6931 up-regulated and 6007 down-regulated genes. A total of 199 up-regulated and 571 down-regulated genes were obtained on taking intersection of DEGs and 1811 IRGs, which were subjected to further analysis as candidate DE-IRGs for stage IV CRC (Figure 1B–D).

### GO enrichment analysis, KEGG pathway enrichment analysis, PPI network construction, and identification of hub genes

**Figure 2:**
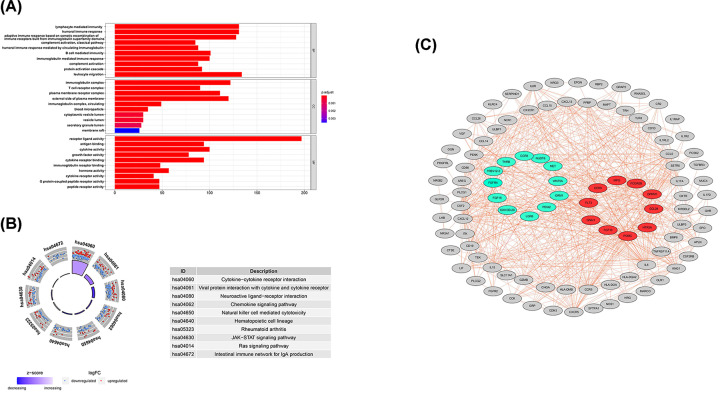
Functional enrichment and the PPI network analyses of the candidate DE-IRGs (**A**) GO analysis. (**B**) KEGG pathway analysis. (**C**) The PPI network of candidate DE-IRGs was constructed by Cytoscape software based on STRING database. Red nodes represented 10 hub genes with the most edges. Green nodes represented 12 genes that were eventually included in the prognostic risk score model.

GO enrichment analysis of the 770 candidate DE-IRGs from stage IV CRC indicated that lymphocyte-mediated immunity, immunoglobulin complex, and receptor ligand activity were the most significantly functionally enriched of BP, CC, and MF, respectively. Cytokine–cytokine receptor interaction was the most relevant pathway identified by KEGG enrichment analysis (Figure 2A,B and Table 2). A PPI network containing 203 nodes and 999 edges was constructed based on these candidate DE-IRGs. The top 30 genes with the largest number of adjacent nodes were visualized. After a comprehensive literature review, 10 of these genes were identified as hub genes, including: *POMC, FLT3, CCR9, FCGR2B, MPO, CCL28, OPRM1, GNAI1, HTR3A*, and *FGF10* (Figure 2C).

**Table 2:** GO and KEGG enrichment analysis of candidate IRGs in IV CRC ranked by *P* value (Top 5)

Categor y	GO or KEGG ID	GO or KEGG term	*P*.adjust	Count
BP	GO:0002449	Lymphocyte-mediated immunity	4.15E-91	131
BP	GO:0006959	Humoral immune response	1.15E-90	131
BP	GO:0002460	Adaptive immune response based on somatic recombination of immune receptors built from immunoglobulin superfamily domains	3.01E-86	128
BP	GO:0006958	Complement activation, classical pathway	1.90E-82	85
BP	GO:0002455	Humoral immune response mediated by circulating immunoglobulin	1.91E-82	88
CC	GO:0019814	Immunoglobulin complex	3.12E-99	122
CC	GO:0042101	T-cell receptor complex	2.74E-98	90
CC	GO:0098802	Plasma membrane receptor complex	1.57E-80	111
CC	GO:0009897	External side of plasma membrane	3.00E-75	120
CC	GO:0042571	Immunoglobulin complex, circulating	1.33E-51	49
MF	GO:0048018	Receptor ligand activity	4.71E-97	197
MF	GO:0003823	Antigen binding	1.67E-94	94
MF	GO:0005125	Cytokine activity	6.20E-86	100
MF	GO:0008083	Growth factor activity	1.18E-68	78
MF	GO:0005126	Cytokine receptor binding	4.80E-65	94
KEGG	hsa04060	Cytokine–cytokine receptor interaction	1.84E-98	136
KEGG	hsa04061	Viral protein interaction with cytokine and cytokine receptor	5.58E-36	50
KEGG	hsa04080	Neuroactive ligand–receptor interaction	4.31E-27	76
KEGG	hsa04062	Chemokine signaling pathway	1.05E-16	45
KEGG	hsa04650	Natural killer cell–mediated cytotoxicity	2.39E-16	37

### Establishment of prognostic risk score model

**Figure 3:**
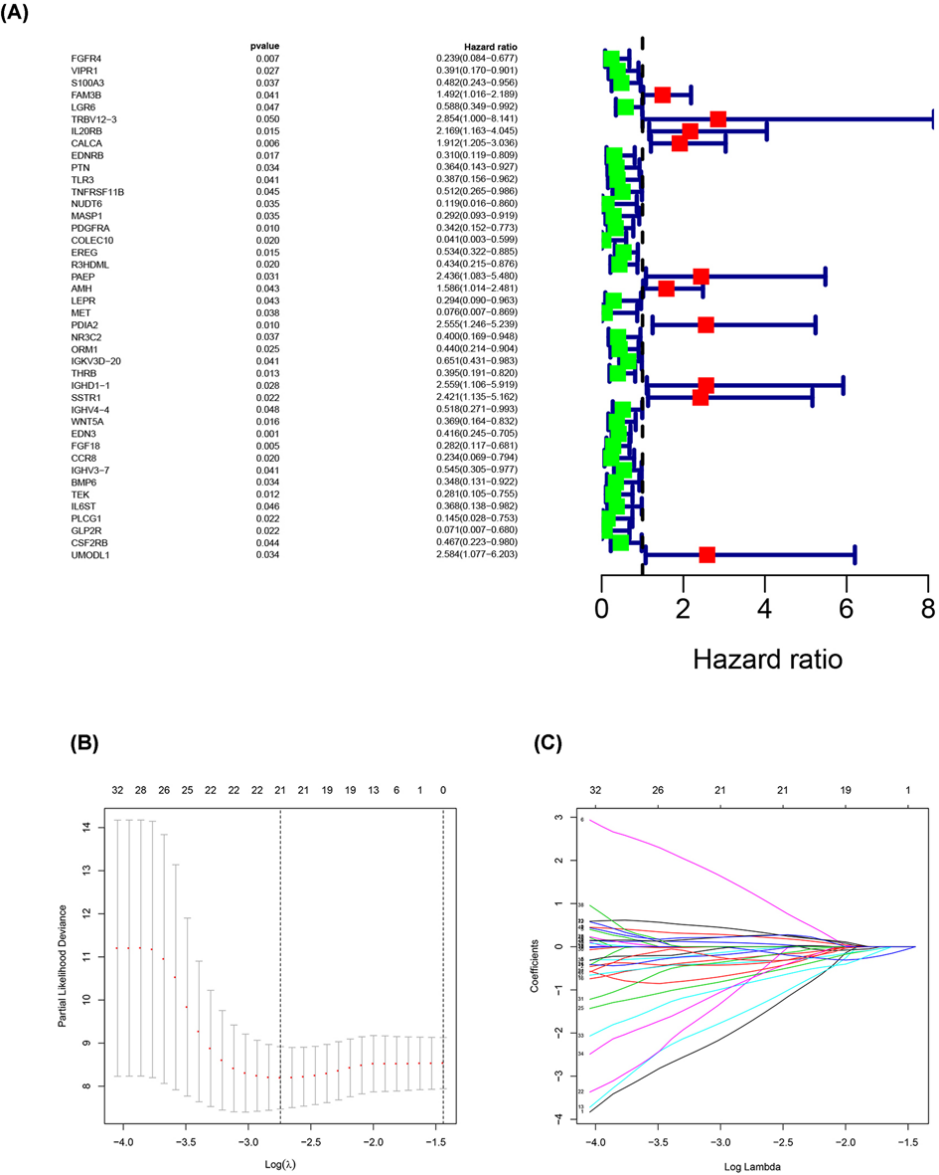
Establishment of prognostic risk score model (**A**) Prognostic values of survival-associated DE-IRGs: Forest plot of survival-associated DE-IRGs. (**B**) Tuning parameter (λ) selection in the LASSO logistic regression used 10,000-fold cross-validation via minimum criteria. The binomial deviance was plotted versus log (λ). (**C**) LASSO coefficient profiles of 63 survival-associated IRGs. A coefficient profile plot was produced versus the log (λ).

As shown in Figure 3, univariate Cox regression analysis was performed using the 770 candidate stage IV CRC DE-IRGs to search for genes related to survival, resulting in identification of 63 genes in total. LASSO was used to further narrow down correlated genes, with 42 genes extracted. Finally, 12 genes were determined as variables for use in construction of the prognostic risk score model by multivariate Cox regression analysis. The calculation formula was as follows:

$$\begin{array}{l} \text{Risk score}=(-4.3781\;\times \text{ Exp}\;FGFR4)+(-0.7465\times \text{Exp}\;LGR6)+(3.9474\times \text{Exp}\;TRBV12\text{-}3)+\\ (-4.8243\times \text{Exp}\;NUDT6)\;+\;(-4.5562\times \text{Exp}\;MET)+(1.1468\;\times \text{ Exp}\;PDIA2)+\\ (-1.5898\times \text{Exp}\;ORM1)+(-1.0328\times \text{Exp}\;IGKV3D\text{-}20\text{)}+(-0.7592\times \text{Exp}\;THRB)+\\ (-1.2434\times \text{Exp}\;WNT5A)+(-2.2298\times \text{Exp}\;FGF18)+(-2.7483\times \text{Exp}\;CCR8\text{)} \end{array}$$

### Validation of the prognostic risk score model

**Figure 4:**
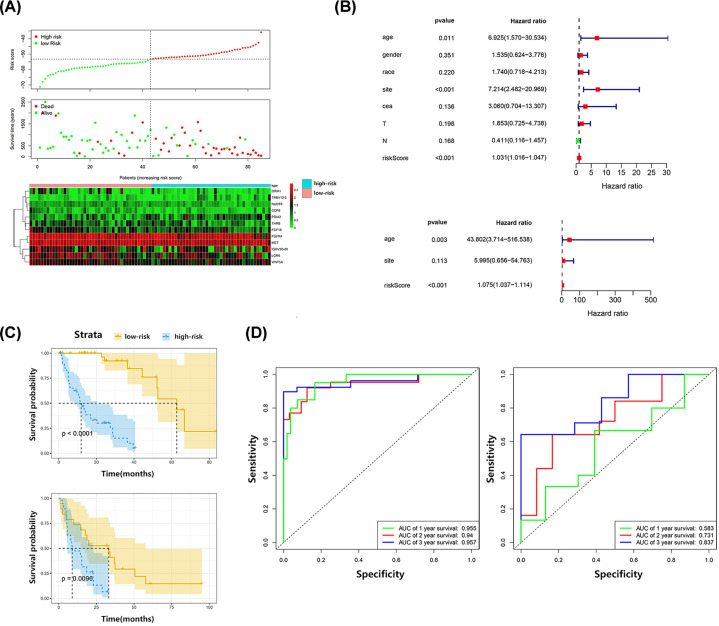
Validation of the prognostic risk score model (**A**) Risk score distribution, survival status, and expression patterns of 12 included genes in risk score model for high- and low-risk patients from training set. (**B**) Univariate and multivariate Cox regression analysis of OS. (**C**) Kaplan–Meier survival curves of high- and low-risk patients in training set and verification set. (**D**) The time dependent ROC at 1-, 2-, and 3-year OS of training set and verification set.

Patient data in the training and validation sets were divided into the high- and low-risk groups, based on their respective median risk scores. Significant survival differences were detected between patients in the high- and low-risk sets in both the training and validation sets (*P*<0.0001, *P*=0.0096, respectively). In the training set, the Harrell’s C-index for survival prediction was 0.701, and the AUC values for 1-, 2-, and 3-year OS were 0.955, 0.940, and 0.957, respectively. In the external validation set, Harrell’s C-index for survival prediction was 0.685, and the AUC values for 1-, 2-, and 3-year OS were 0.583, 0.731, and 0.837, respectively. Univariate and multivariate regression analyses were carried out by adding the factor of risk score in the training set. Only age and risk score were independent predictive factors for OS (*P*=0.003 and *P*<0.001, respectively) (Figure 4).

### Immune-related characteristics of high- and low-risk patients, based on the prognostic risk score model

**Figure 5:**
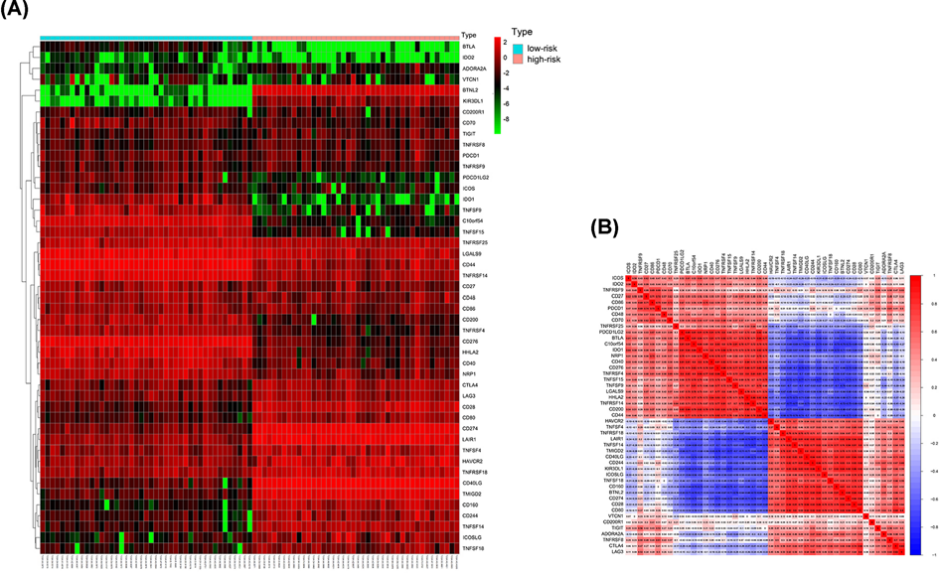
Relationship between the risk score and ICGs (**A**) The heatmap of ICGs between high- and low-risk patients. (**B**) Correlation analyses of pairwise relationships in the expression of ICGs.

**Figure 6:**
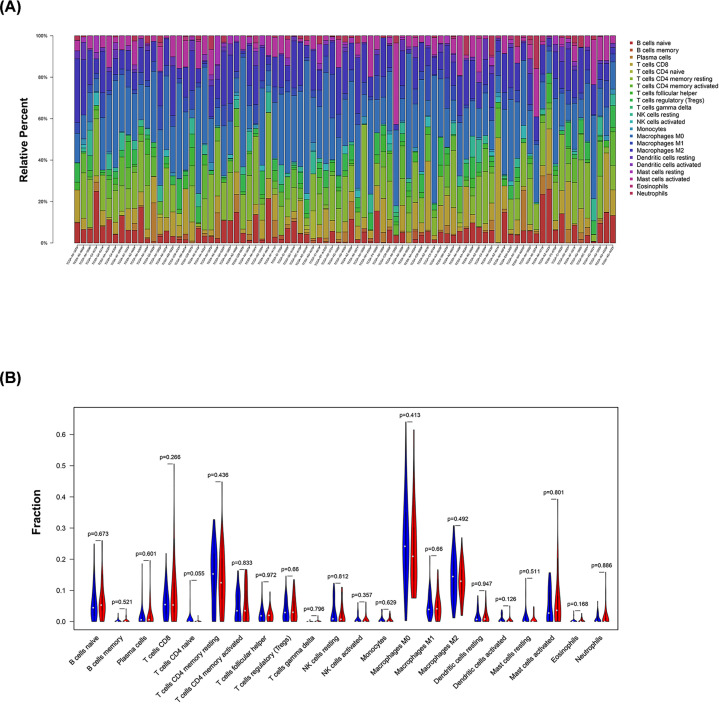
Landscape of immune infiltration in high- and low-risk patients with stage IV CRC (**A**) Bar plots visualizing relative proportion of 22 immune cells in high- and low-risk patients. (**B**) Violin plots visualizing significantly different immune cell infiltrations between high- and low- risk patients.

Except for *CD200R1, PDCD1, TIGIT, TNFRSF9*, and *VTCN1* (*P*=0.541, 0.098, 0.095, 0.527, and 0.459, respectively), all of the other 42 ICGs showed significant differences in gene expression between high- and low-risk patients (Figure 5). Due to the significant relationship between risk score and ICGs, all 12 genes included in the model were individually analyzed in the TISIDB database. A significant relationship was detected between *FGFR4, LGR6, PDIA2, ORM1*, and *WNT5A* expression differences and treatment response in immunotherapy clinical trials (Table 3). However, there was no significant difference in the proportion of the 22 immune cell types analyzed between high- and low-risk patients (Figure 6).

**Table 3:** The relationship between the expression of IRGs included in this prognostic risk score model and the clinical outcome of immunotherapy (TISIDB)

Gene symbol	PMID	Cancer type	Group	Drug	The number of resp onders	The number of non- responders	Log2 (fold change)	*P* value
FGFR4	29033 130	Melanoma	All	Anti-PD-1 (Nivolumab)	26	23	1.409	0.017
LGR6	29301 960	Clear cell renal cell carcinoma (ccRCC)	All	Anti-PD-1 (Nivolumab)	4	8	1.065	0.043
LGR6	29301 960	Clear cell renal cell carcinoma (ccRCC)	Non-VEG FRi	Anti-PD-1 (Nivolumab)	2	8	1.602	0.019
PDIA2	26997 480	Melanoma	MAP Ki	Anti-PD-1 (Pembrolizumab and Nivolumab)	6	5	-1.229	0.050
ORM1	29443 960	Urothelial cancer	All	Anti-PD-L1 (Atezolizumab)	68	230	-1.045	0.038
WNT5 A	26997 480	Melanoma	All	Anti-PD-1 (Pembrolizumab and Nivolumab)	14	12	-1.048	0.028
WNT5 A	26997 480	Melanoma	MAP Ki	Anti-PD-1 (pembrolizu mab and nivolumab)	6	5	-2.179	0.006

## Discussion

Stage IV CRC is commonly referred to as metastatic colorectal cancer (MCRC) and the overall 5-year survival rate for patients with this condition is 40–64% [20,21]. Autopsies demonstrated that liver metastases are the leading cause of death in such patients [22]. In 2016, ESMO proposed the concept of ‘no evidence of disease (NED)’ to the diagnosis and treatment strategy for MCRC, representing a break from the traditional understanding of stage IV CRC [23]. In recent years, the efficacy of drugs such as trifluridine, tipiracil, bevacizumab, cetuximab, regorafenib, encorafenib, nivolumab, and pembrolizumab has been evaluated and confirmed in different subgroups of patients with stage IV CRC [24–29]. OS of these patients is expected to be extended in the future and it is essential to make prognostic predictions, to individualize their treatment plans. Nevertheless, no such method is available in clinical practice, with only stage II and overall CRC previously investigated in this research field [5–8]. While only a few prognostic factors for stage IV CRC have been reported [30,31]. In the present study, we constructed a prognostic risk score model for patients with stage IV CRC, based on IRGs, as well as screening for immune-related hub genes during the construction process. *POMC, CCR9, FCGR2B, CCL28, OPRM1, GNAI1, HTR3A*, and *FGF10* are all genes rarely reported in CRC research. POMC has a role in tumor development, by promoting epithelial–mesenchymal transition (EMT) [32–34]. It also has been reported as an independent prognostic factor in surgically resected non-small cell lung cancer [35]. CCR9/CCL25 also has an important role in tumor invasion and metastasis by promoting EMT in liver, non-small cell lung cancer, and breast cancer cell lines [36,37]. More interestingly, intratumoral delivery of CCL25 can enhance PD-L1/PD-1 antibody therapy against triple-negative breast cancer by recruiting CCR9+ T cells [38]. Regarding CCL28, recent studies have found that blocking of β-catenin–CCL28 can reduce Treg cell infiltration and inhibit tumor growth. Therefore, the β-catenin–CCL28–Treg cell axis may be involved in immunosuppression [39]. FGF10 promotes tumor invasion and metastasis by binding to FGFR2 in pancreatic cancer and breast cancer cell lines [40,41]. Of the 12 genes included in the prognostic risk score model, only *FGFR4, MET, WNT5A*, and *CCR8* have been studied in relation to tumor immunity. Specifically, FGFR4 can act directly on tumor cells to influence paracrine signaling, angiogenesis, and immune escape, normalizing the tumor microenvironment [42]. MET is a tumor-associated antigen facilitating tumor cell recognition by CD8+ cytotoxic T cells, which triggers immune system activation [43]. WNT5A has dual effects on the tumor microenvironment; it can promote inflammation and chemotaxis of immune cells through activation of the ROR1/Akt/P65 pathway, as well as stimulating the TLR/MyD88/p50 pathway in myelomonocytic cells, to promote synthesis of the anti-inflammatory cytokine, IL-10 [44]. Further, Tregs positive for CCR8 expression are major drivers of immune regulation, and CCL1 can induce CCR8 expression, thus enhancing the suppressive function [45]. The effect of these genes in the development and progression of CRC warrant further study, particularly in the context of tumor induced immune tolerance.

The prognostic risk score model developed in the present study included 12 IRGs. In the training set, Harrell’s C-index for survival prediction was 0.701, with AUC values for 1-, 2-, and 3-year OS of 0.955, 0.940, and 0.957, respectively. In the external validation set, Harrell’s C-index for survival prediction was 0.685, with AUC values for 1-, 2-, and 3-year OS of 0.583, 0.731, and 0.837, respectively. Compared with the three published prognostic models for overall CRC based on IRGs, this model contains completely different genes and has good predictive power, particularly for 2- and 3-year OS [46–48]. In addition, patients classified as high- and low-risk according to this model showed significant differences in ICGs expression. We further explored all 12 genes in this model in the TISIDB database and found that differential expression of *LGR6, PDIA2, ORM1, FGFR4*, and *WNT5A* were associated with treatment response to immunotherapy in past clinical trials. These findings imply that our risk score model potentially has the ability to predict immunotherapy responses. Further, the relationship between the genes included in this model and current anti-PD1 and anti-PDL1 treatments warrants further exploration.

Other researchers have constructed CRC prognostic risk model based on DNA methylation markers, autophagy-related genes, and hypoxia-related genes, among others [49–51], and most generated acceptable results; however, no perfect prognostic model has been achieved, due to individual differences among patients and unknown disease pathogenesis. For patients with stage IV CRC, it will be necessary to construct additional prognostic risk models in the future, based on different methods and perspectives. In recent years, the important role of the tumor immune microenvironment in the occurrence and development of malignant tumors has attracted wide attention. Since both tumor and host are directly affected by genomic factors, it is feasible to predict immunotherapy responses and prognosis of patients with CRC, based on ICGs. To our knowledge, this is the first prognostic risk score model for patients with stage IV CRC. Furthermore, the predictive power of this model could be further improved by inclusion of relevant clinical parameters.

The limitations of the present study include its reliance on online data analysis and relatively small sample size. More in-depth analyses could not be conducted in the present study, due to the lack of basic experiments. Consequently, the predictive power of this risk score model requires further verification in multi-center and prospective research investigations.

## Conclusions

The prognostic risk score model for stage IV CRC developed in the present study based on immune-related genes has acceptable predictive power, and is closely related to the expression of ICGs.

## Supplementary Material

Supplementary Table S1

## Data Availability

The datasets generated and analyzed during the current study are available in TCGA official website repository (https://portal.gdc.cancer.gov/) and Gene Expression Omnibus (GEO) (https://www.ncbi.nlm.nih.gov/geo/).

## Authors' Information

The corresponding author (Ke Xu) would be contacted if necessary.

## Abbreviations

AUC: area under the curve
CEA: carcinoembryonic antigen
CRC: colorectal cancer
DEG: differentially expressed gene
DE-IRG: differentially expressed immune-related gene
EMT: epithelial–mesenchymal transition
ESMO: European society for medical oncology
FC: fold changes
GEO: Gene Expression Omnibus
GO: Gene Ontology
ICG: immune checkpoint coding gene
IRG: immune-related gene
KEGG: Kyoto Encyclopedia of Genes and Genomes
MCRC: metastatic colorectal cancer
NCCN: national comprehensive cancer network
OS: overall survival
PPI: protein–protein interaction
ROC: receiver operating characteristic
TCGA: The Cancer Genome Atlas

## References

[1] Siegel R.L., Miller K.D. and Jemal A. (2020) Cancer statistics, 2020. CA Cancer J. Clin. 70, 7–30 10.3322/caac.21590 31912902

[2] Lee W.S., Yun S.H., Chun H.K. et al. (2007) Pulmonary resection for metastases from colorectal cancer: prognostic factors and survival. Int. J. Colorectal Dis. 22, 699–704 10.1007/s00384-006-0218-2 17109105

[3] Van Cutsem E., Nordlinger B., Adam R. et al. (2006) Towards a pan-European consensus on the treatment of patients with colorectal liver metastases. Eur. J. Cancer 42, 2212–2221 10.1016/j.ejca.2006.04.012 16904315

[4] Yoo P.S., Lopez-Soler R.I., Longo W.E. and Cha C.H. (2006) Liver resection for metastatic colorectal cancer in the age of neoadjuvant chemotherapy and bevacizumab. Clin. Colorectal Cancer 6, 202–207 10.3816/CCC.2006.n.036 17026789

[5] Benson A.B. III, Schrag D., Somerfield M.R. et al. (2004) American Society of Clinical Oncology recommendations on adjuvant chemotherapy for stage II colon cancer. J. Clin. Oncol. 22, 3408–3419 10.1200/JCO.2004.05.063 15199089

[6] Figueredo A., Charette M.L., Maroun J., Brouwers M.C. and Zuraw L. (2004) Adjuvant therapy for stage II colon cancer: a systematic review from the Cancer Care Ontario Program in evidence- based care's gastrointestinal cancer disease site group. J. Clin. Oncol. 22, 3395–3407 10.1200/JCO.2004.03.087 15199087

[7] Gill S., Loprinzi C.L., Sargent D.J. et al. (2004) Pooled analysis of fluorouracil-based adjuvant therapy for stage II and III colon cancer: who benefits and by how much? J. Clin. Oncol. 22, 1797–1806 10.1200/JCO.2004.09.059 15067028

[8] Shi M. and He J. (2016) ColoFinder: a prognostic 9-gene signature improves prognosis for 871 stage II and III colorectal cancer patients. PeerJ 4, e1804 10.7717/peerj.1804 26989635 PMC4793313

[9] Gentles A.J., Newman A.M., Liu C.L. et al. (2015) The prognostic landscape of genes and infiltrating immune cells across human cancers. Nat. Med. 21, 938–945 10.1038/nm.3909 26193342 PMC4852857

[10] Chaudhary B. and Elkord E. (2016) Regulatory T Cells in the Tumor Microenvironment and Cancer Progression: Role and Therapeutic Targeting. Vaccines (Basel) 4, 27509527 10.3390/vaccines4030028 PMC5041022

[11] Coto-Llerena M., Ercan C., Kancherla V. et al. (2020) High Expression of FAP in Colorectal Cancer Is Associated With Angiogenesis and Immunoregulation Processes. Front. Oncol. 10, 979 10.3389/fonc.2020.00979 32733792 PMC7362758

[12] Troncone E. and Monteleone G. (2019) Smad7 and Colorectal Carcinogenesis: A Double-Edged Sword. Cancers (Basel) 11, 612 10.3390/cancers11050612 PMC6563107 31052449

[13] Ritchie M.E., Phipson B., Wu D. et al. (2015) limma powers differential expression analyses for RNA- sequencing and microarray studies. Nucleic Acids Res. 43, e47 10.1093/nar/gkv007 25605792 PMC4402510

[14] Yu G., Wang L.G., Han Y. and He Q.Y. (2012) clusterProfiler: an R package for comparing biological themes among gene clusters. OMICS 16, 284–287 10.1089/omi.2011.0118 22455463 PMC3339379

[15] von Mering C., Huynen M., Jaeggi D., Schmidt S., Bork P. and Snel B. (2003) STRING: a database of predicted functional associations between proteins. Nucleic Acids Res. 31, 258–261 10.1093/nar/gkg034 12519996 PMC165481

[16] Shannon P., Markiel A., Ozier O. et al. (2003) Cytoscape: a software environment for integrated models of biomolecular interaction networks. Genome Res. 13, 2498–2504 10.1101/gr.1239303 14597658 PMC403769

[17] Gui J. and Li H. (2005) Penalized Cox regression analysis in the high-dimensional and low-sample size settings, with applications to microarray gene expression data. Bioinformatics 21, 3001–3008 10.1093/bioinformatics/bti422 15814556

[18] Newman A.M., Liu C.L., Green M.R. et al. (2015) Robust enumeration of cell subsets from tissue expression profiles. Nat. Methods 12, 453–457 10.1038/nmeth.3337 25822800 PMC4739640

[19] Ru B., Wong C.N., Tong Y. et al. (2019) TISIDB: an integrated repository portal for tumor-immune system interactions. Bioinformatics 35, 4200–4202 10.1093/bioinformatics/btz210 30903160

[20] Marin C., Robles R., Lopez Conesa A., Torres J., Flores D.P. and Parrilla P. (2013) Outcome of strict patient selection for surgical treatment of hepatic and pulmonary metastases from colorectal cancer. Dis. Colon Rectum 56, 43–50 10.1097/DCR.0b013e3182739f5e 23222279

[21] Adam R., Hoti E. and Bredt L.C. (2010) Evolution of neoadjuvant therapy for extended hepatic metastases–have we reached our (non-resectable) limit? J. Surg. Oncol. 102, 922–931 10.1002/jso.21727 21165994

[22] Foster J.H. (1984) Treatment of metastatic disease of the liver: a skeptic's view. Semin. Liver Dis. 4, 170–179 10.1055/s-2008-1040656 6205450

[23] Van Cutsem E., Cervantes A., Adam R. et al. (2016) ESMO consensus guidelines for the management of patients with metastatic colorectal cancer. Ann. Oncol. 27, 1386–1422 10.1093/annonc/mdw235 27380959

[24] Mayer R.J., Van Cutsem E., Falcone A. et al. (2015) Randomized trial of TAS-102 for refractory metastatic colorectal cancer. N. Engl. J. Med. 372, 1909–1919 10.1056/NEJMoa1414325 25970050

[25] Venook A.P., Niedzwiecki D., Lenz H.J. et al. (2017) Effect of First-Line Chemotherapy Combined With Cetuximab or Bevacizumab on Overall Survival in Patients With KRAS Wild-Type Advanced or Metastatic Colorectal Cancer: A Randomized Clinical Trial. JAMA 317, 2392–2401 10.1001/jama.2017.7105 28632865 PMC5545896

[26] Grothey A., Van Cutsem E., Sobrero A. et al. (2013) Regorafenib monotherapy for previously treated metastatic colorectal cancer (CORRECT): an international, multicentre, randomised, placebo-controlled, phase 3 trial. Lancet 381, 303–312 10.1016/S0140-6736(12)61900-X 23177514

[27] Le D.T., Durham J.N., Smith K.N. et al. (2017) Mismatch repair deficiency predicts response of solid tumors to PD-1 blockade. Science 357, 409–413 10.1126/science.aan6733 28596308 PMC5576142

[28] Overman M.J., McDermott R., Leach J.L. et al. (2017) Nivolumab in patients with metastatic DNA mismatch repair-deficient or microsatellite instability-high colorectal cancer (CheckMate 142): an open-label, multicentre, phase 2 study. Lancet Oncol. 18, 1182–1191 10.1016/S1470-2045(17)30422-9 28734759 PMC6207072

[29] Overman M.J., Lonardi S., Wong K.Y.M. et al. (2018) Durable Clinical Benefit With Nivolumab Plus Ipilimumab in DNA Mismatch Repair-Deficient/Microsatellite Instability-High Metastatic Colorectal Cancer. J. Clin. Oncol. 36, 773–779 10.1200/JCO.2017.76.9901 29355075

[30] Moik F., Posch F., Grilz E. et al. (2020) Haemostatic biomarkers for prognosis and prediction of therapy response in patients with metastatic colorectal cancer. Thromb. Res. 187, 9–17 10.1016/j.thromres.2020.01.002 31945589 PMC7212083

[31] Zhang L., Zhang J., Wang Y. et al. (2019) Potential prognostic factors for predicting the chemotherapeutic outcomes and prognosis of patients with metastatic colorectal cancer. J. Clin. Lab. Anal. 33, e22958 10.1002/jcla.22958 31218745 PMC6805281

[32] Tsai H.E., Liu L.F., Dusting G.J. et al. (2012) Pro-opiomelanocortin gene delivery suppresses the growth of established Lewis lung carcinoma through a melanocortin-1 receptor- independent pathway. J. Gene Med. 14, 44–53 10.1002/jgm.1625 22147647

[33] Fassnacht M., Hahner S., Hansen I.A. et al. (2003) N-terminal proopiomelanocortin acts as a mitogen in adrenocortical tumor cells and decreases adrenal steroidogenesis. J. Clin. Endocrinol. Metab. 88, 2171–2179 10.1210/jc.2002-021318 12727972

[34] Herraiz C., Journe F., Abdel-Malek Z., Ghanem G., Jimenez-Cervantes C. and Garcia-Borron J.C. (2011) Signaling from the human melanocortin 1 receptor to ERK1 and ERK2 mitogen-activated protein kinases involves transactivation of cKIT. Mol. Endocrinol. 25, 138–156 10.1210/me.2010-0217 21084381 PMC3089036

[35] Hao L., Zhao X., Zhang B., Li C. and Wang C. (2015) Positive expression of pro-opiomelanocortin (POMC) is a novel independent poor prognostic marker in surgically resected non-small cell lung cancer. Tumour Biol. 36, 1811–1817 10.1007/s13277-014-2784-1 25377161

[36] Niu Y., Tang D., Fan L., Gao W. and Lin H. (2020) CCL25 promotes the migration and invasion of non- small cell lung cancer cells by regulating VEGF and MMPs in a CCR9-dependent manner. Exp. Ther. Med. 19, 3571–3580 32346420 10.3892/etm.2020.8635 PMC7185084

[37] Zhang Z., Sun T., Chen Y. et al. (2016) CCL25/CCR9 Signal Promotes Migration and Invasion in Hepatocellular and Breast Cancer Cell Lines. DNA Cell Biol. 35, 348–357 10.1089/dna.2015.3104 27008282

[38] Chen H., Cong X., Wu C. et al. (2020) Intratumoral delivery of CCL25 enhances immunotherapy against triple-negative breast cancer by recruiting CCR9(+) T cells. Sci. Adv. 6, eaax4690 10.1126/sciadv.aax4690 32064335 PMC6989134

[39] Ji L., Qian W., Gui L. et al. (2020) Blockade of beta-Catenin-Induced CCL28 Suppresses Gastric Cancer Progression via Inhibition of Treg Cell Infiltration. Cancer Res. 10.1158/0008-5472.CAN-19-3074 32156780

[40] Nomura S., Yoshitomi H., Takano S. et al. (2008) FGF10/FGFR2 signal induces cell migration and invasion in pancreatic cancer. Br. J. Cancer 99, 305–313 10.1038/sj.bjc.6604473 18594526 PMC2480967

[41] Abolhassani A., Riazi G.H., Azizi E. et al. (2014) FGF10: Type III Epithelial Mesenchymal Transition and Invasion in Breast Cancer Cell Lines. J. Cancer 5, 537–547 10.7150/jca.7797 25057305 PMC4107230

[42] Katoh M. (2016) FGFR inhibitors: Effects on cancer cells, tumor microenvironment and whole- body homeostasis (Review). Int. J. Mol. Med. 38, 3–15 10.3892/ijmm.2016.2620 27245147 PMC4899036

[43] Schag K., Schmidt S.M., Muller M.R. et al. (2004) Identification of C-met oncogene as a broadly expressed tumor-associated antigen recognized by cytotoxic T-lymphocytes. Clin. Cancer Res. 10, 3658–3666 10.1158/1078-0432.CCR-03-0640 15173072

[44] Lopez-Bergami P. and Barbero G. (2020) The emerging role of Wnt5a in the promotion of a pro- inflammatory and immunosuppressive tumor microenvironment. Cancer Metastasis Rev. 39, 933–952 10.1007/s10555-020-09878-7 32435939

[45] Karin N. (2018) Chemokines and cancer: new immune checkpoints for cancer therapy. Curr. Opin. Immunol. 51, 140–145 10.1016/j.coi.2018.03.004 29579623

[46] Lu Y., Zhou X., Liu Z., Wang B., Wang W. and Fu W. (2020) Assessment for Risk Status of Colorectal Cancer Patients: A Novel Prediction Model Based on Immune-Related Genes. DNA Cell Biol. 39, 958–964 10.1089/dna.2019.5195 32243216

[47] Ge P., Wang W., Li L. et al. (2019) Profiles of immune cell infiltration and immune-related genes in the tumor microenvironment of colorectal cancer. Biomed. Pharmacother. 118, 109228 10.1016/j.biopha.2019.109228 31351430

[48] Bai J., Zhang X., Xiang Z.X., Zhong P.Y. and Xiong B. (2020) Identification of prognostic immune-related signature predicting the overall survival for colorectal cancer. Eur. Rev. Med. Pharmacol. Sci. 24, 1134–1141 32096169 10.26355/eurrev_202002_20164

[49] Deng Y., Wan H., Tian J. et al. (2020) CpG-methylation-based risk score predicts progression in colorectal cancer. Epigenomics 12, 605–615 10.2217/epi-2019-0300 32180433

[50] Huang Z., Liu J., Luo L. et al. (2019) Genome-Wide Identification of a Novel Autophagy-Related Signature for Colorectal Cancer. Dose Response 17, 1559325819894179 10.1177/1559325819894179 31853237 PMC6906358

[51] Lee J.H., Jung S., Park W.S. et al. (2019) Prognostic nomogram of hypoxia-related genes predicting overall survival of colorectal cancer-Analysis of TCGA database. Sci. Rep. 9, 1803 10.1038/s41598-018-38116-y 30755640 PMC6372658

